# The Interactive Effects of Age and *PICALM* rs541458 Polymorphism on Cognitive Performance, Brain Structure, and Function in Non-demented Elderly

**DOI:** 10.1007/s12035-016-0358-5

**Published:** 2017-01-23

**Authors:** Zhen Liu, Xiangwei Dai, Junying Zhang, Xin Li, Yaojing Chen, Chao Ma, Kewei Chen, Dantao Peng, Zhanjun Zhang

**Affiliations:** 1 State Key Laboratory of Cognitive Neuroscience and Learning & IDG/McGovern Institute for Brain Research, Beijing Normal University, Beijing, 100875 People’s Republic of China; 2 BABRI Centre, Beijing Normal University, Beijing, 100875 People’s Republic of China; 3 Banner Alzheimer’s Institute, Phoenix, AZ 85006 USA; 4 Department of Neurology, China-Japan Friendship Hospital, Beijing, 100029 People’s Republic of China

**Keywords:** Alzheimer’s disease, Executive function, Functional connectivity, Gray matter volume, *PICALM*

## Abstract

The *PICALM* rs541458 T allele has been recognized as a risk factor for late-onset Alzheimer’s disease, and age might modulate the effects that genetic factors have on cognitive functions and brain. Thus, the current study intended to examine whether the effects of rs541458 on cognitive functions, brain structure, and function were modulated by age in non-demented Chinese elderly. We enrolled 638 subjects aged 50 to 82 years and evaluated their cognitive functions through a series of neuropsychological tests. Seventy-eight of these participants also received T1-weighted structural and resting state functional magnetic resonance imaging. Dividing subjects into groups <65 and ≥65 years old, results of neuropsychological tests showed that interactive effects of rs541458 × age existed with regard to executive function and processing speed after controlling for gender, years of education and *APOE* ε4 status. In addition, the effects of rs541458 on resting state functional connectivity of left superior parietal gyrus within left frontal-parietal network and on gray matter volume of left middle temporal gyrus were modulated by age. Furthermore, reduction of functional connectivity of left superior parietal gyrus was closely related with better executive function in the T allele carriers <65 years old. Further, greater volume of left middle temporal gyrus was significantly related to better executive function in both CC genotype <65 years old and CC genotype ≥65 years old groups, separately. Pending further confirmation from additional studies, our results support the hypothesis that the modulation of age, with respect to the rs541458, has interactional effects on cognitive performance, brain function, and structural measurements.

**Electronic supplementary material:**

The online version of this article (doi:10.1007/s12035-016-0358-5) contains supplementary material, which is available to authorized users.

## Introduction

Alzheimer’s disease (AD) is the most common type of dementia. Due to the sharp increase in the number of patients [1] and the high mortality rate [2], AD is currently an important global health problem. Among many risk factors, increased age has always been a pivotal risk factor for late-onset AD (LOAD) [3]. In fact, the prevalence of AD increases with age [4], almost doubling every 5 years after the age of 60 [5].

In addition to age, several well-recognized genetic factors augment the complexity of LOAD [6]. For instance, the apolipoprotein E (*APOE*) ε4 allele is the best known genetic LOAD risk factor, which is associated with increased incidence and a decreased age of onset of LOAD [7]. It has been suggested that the ε4 allele would likely account for approximately 50% or more of the LOAD cases in the USA [8, 9]. Large-scale genome-wide association studies (GWAS) have been carried out to locate additional susceptible loci to more fully understand the genetic etiology of LOAD. Among these loci is the one encoding the -binding clathrin assembly protein (PICALM) [10]. The 112 kb *PICALM* gene is located on chromosome 11q14. In the central nervous system, PICALM has primarily been identified in neurons, astrocytes, and oligodendrocytes [11]. Evidence has shown that PICALM plays a major role in clathrin-mediated endocytosis [12], in which it recruits clathrin and adaptor protein 2 to the plasma membrane and transports target proteins to be processed in lysosomes or endosomes [12, 13]. Thus, PICALM would be further associated with amyloid precursor protein (APP) metabolism [14, 15], which is hypothesized to play a key role in AD pathogenesis.

The SNP rs541458 is located 8 kb upstream of the *PICALM* gene [16], with C as the minor allele and T as the major allele. An early GWAS study of Europeans found the rs541458 polymorphism to be a susceptible site for LOAD [17]. Subsequently, Lambert et al. verified the risk of the T allele of this SNP in a meta-analysis by examining three different European populations [18]. Other studies of Caucasians also reported the association between the rs541458 T allele and the LOAD risk [19, 20]. However, the LOAD risk associated with the rs541458 polymorphism was not duplicated in samples from the southern China region [21]. These results suggest that the effects of the rs541458 polymorphism might not be consistent among different ethnic groups.

Because of the crucial role of the brain in aging biology [22], many groups have investigated the patterns of brain aging with respect to brain structure and function [23, 24]. Additionally, the effects of a specific gene such as *PICALM* on the brain are not static but are dynamic and aging-related [25], indicating that age would modulate the effects of genetic factors in the brain. A previous study by Sweet et al. showed that the rs541458 T allele was associated with an earlier age at the midpoint of general cognitive function decline [26]. However, few studies have referred to the regulating effects of age on the rs541458 SNP based on brain measures. Taking 65 years of age or older as the onset age of LOAD [27], we divided the elderly into young (<65 years old) and old (≥65 years old) groups to assess the age modulation effects.

Indeed, cognitive deficits are often thought to be associated with brain functional and/or structural impairments [28, 29], which can be measured by using volumetric or functional magnetic resonance imaging (MRI) neuroimaging techniques. For example, resting state blood-oxygen-level dependent (BOLD) MRI allowed the delineation of the human neural functional architecture into multinetworks, such as the default mode network (DMN) and frontal-parietal network (FPN) [30]. Some studies have highlighted a number of functional networks showing significant age-related changes, including in the DMN and left FPN [31, 32]. The *APOE* disrupted the brain network connectivity, including the connectivity of both the posterior [33, 34] and anterior [35] DMNs as well as the left FPN [36]. Additionally, the risk allele (i.e., G allele) of the *PICALM* rs3851179 polymorphism was associated with weaker negative functional connectivity between the left hippocampus and the right precuneus and between the right hippocampus and the left superior frontal gyrus [37]. By using T1-weighted volumetric MRI to assess brain structures, a previous study suggested that atrophy was primarily located in the temporal and parietal regions in normal aging [38]. A recent study demonstrated that rs3851179 had a nominally significant main effect on hippocampal volume in healthy, young subjects [37], although negative findings have been reported in the elderly [39]. This may suggest that age would modulate the association between heredity and the human brain.

For the rs541458 SNP, we feel that relatively little is known about its effects on the resting state functional connectivity and whole brain gray matter volumes as well as neuropsychological performance (see the study by Sweet et al. [26]) and, more importantly, whether age could modulate such effects. Although it is weak, the existing evidence for *PICALM* itself and for genetic risk factors motivated us to make the following hypothesis: the effects of the *PICALM* rs541458 polymorphism on cognitive function, functional networks, and gray matter volumes are modulated by age. In the present study, we first examined such an age moderation effect on neuropsychological performance in a larger sample of non-demented Chinese elderly. Second, we investigated this modulated effect of age on functional connectivity and gray matter volumes in an imaging sub-cohort.

## Materials and Methods

### Participants

The subjects were from the Beijing Aging Brain Rejuvenation Initiative (BABRI) Study Group, which is an ongoing longitudinal study examining the brain and cognitive decline in an elderly, community-dwelling population [40]. All enrolled participants were Han Chinese. Participants were qualified for our study if they met the following criteria: right-handed and native Chinese speakers, no less than 50 years old, at least 6 years of education, no history of neurological or psychiatric disorders, and could provide a successful blood sample for the genotyping analysis. For this *PICALM* investigation, we only included individuals who were “clinically non-demented,” as determined by using the DSM IV, Petersen’s dementia criteria, and Clinical Dementia Rating (score = 0). Accordingly, a total of 638 subjects (aged 50–82 years) were included in the present study. To investigate the age modulation effects, the subjects were divided into young (<65 years old) and old (≥65 years old) groups. The demographic information for each group is presented in Table 1. The study was approved by the Institutional Review Board of the Beijing Normal University Imaging Center for Brain Research. Written informed consent was obtained from each participant.

**Table 1:** Demographics and neuropsychological test results of all participants

	Age <65 (*N* = 376)		Age ≥65 (*N* = 262)		*F*/*χ* ^2^_age × *PICALM*_	*F*/*χ* ^2^_age_	*F*/*χ* ^2^_*PICALM*_
	*PICALM* CC (*N* = 87)	*PICALM* T carriers (*N* = 289)	*PICALM* CC (*N* = 64)	*PICALM* T carriers (*N* = 198)	(*P*)	(*P*)	(*P*)
Sex (male/female)	34/53	77/212	27/37	83/115	2.064 (0.151)	0.377 (0.539)	3.640 (0.056)
Education	10.01 ± 2.52	10.56 ± 2.68	12.22 ± 3.57	12.07 ± 3.55	1.455 (0.228)	41.746 (<0.001)*	0.491 (0.484)
*APOE* ε4 or not	16/71	44/245	7/57	42/156	3.483 (0.062)	2.608 (0.106)	2.540 (0.111)
General mental status							
MMSE	27.92 ± 1.71	28.00 ± 1.79	27.83 ± 1.57	27.32 ± 2.05	1.968 (0.161)	14.372 (<0.001)*	1.553 (0.213)
Memory function							
AVLT-delay	5.98 ± 2.69	6.18 ± 2.50	5.00 ± 2.20	4.94 ± 2.56	0.002 (0.965)	37.505 (<0.001)*	0.004 (0.951)
AVLT-total	31.52 ± 9.22	32.00 ± 9.07	27.56 ± 8.56	27.64 ± 9.50	0.144 (0.705)	38.705 (<0.001)*	0.004 (0.947)
ROCF-delay	14.13 ± 5.74	14.42 ± 6.63	11.64 ± 6.61	11.78 ± 5.93	0.020 (0.887)	39.237 (<0.001)*	0.136 (0.713)
Forward digit span	7.47 ± 1.30	7.49 ± 1.39	7.16 ± 1.22	7.26 ± 1.23	0.360 (0.549)	17.927 (<0.001)*	0.186 (0.667)
Backward digit span	4.46 ± 1.30	4.32 ± 1.25	4.20 ± 1.32	4.17 ± 1.31	0.381 (0.538)	11.963 (0.001)*	0.500 (0.480)
Spatial processing							
ROCF-copy	33.44 ± 3.61	33.46 ± 3.37	32.61 ± 5.53	32.75 ± 4.17	0.190 (0.663)	16.033 (<0.001)*	0.115 (0.735)
CDT	24.82 ± 3.32	24.91 ± 3.39	24.36 ± 4.22	24.34 ± 3.70	0.006 (0.936)	13.696 (<0.001)*	0.002 (0.966)
Language							
CVFT	44.37 ± 8.46	46.46 ± 8.47	43.34 ± 9.22	43.41 ± 8.88	0.847 (0.358)	14.411 (<0.001)*	1.349 (0.246)
BNT	23.59 ± 3.64	23.49 ± 3.47	22.64 ± 3.77	22.61 ± 3.78	<0.001 (0.994)	31.019 (<0.001)*	0.007 (0.932)
Processing speed							
SDMT	36.41 ± 11.16	38.62 ± 10.09	31.47 ± 12.05	29.64 ± 9.39	2.342 (0.126)	97.771 (<0.001)*	0.004 (0.949)
TMTa time (s)	58.32 ± 22.66	52.96 ± 16.82	61.86 ± 22.64	66.06 ± 23.85	4.871 (0.028)*	31.481 (<0.001)*	0.102 (0.749)
Executive function							
SCWT C-B time (s)	38.02 ± 14.21	35.21 ± 14.74	41.80 ± 18.19	44.80 ± 20.66	2.693 (0.101)	15.134 (<0.001)*	0.036 (0.850)
SCWT-C time (s)	76.78 ± 18.70	70.97 ± 17.62	81.91 ± 22.33	87.41 ± 23.58	7.033 (0.008)*	33.640 (<0.001)*	0.056 (0.814)
TMTb time (s)	174.83 ± 72.24	153.33 ± 48.97	195.23 ± 77.07	208.87 ± 79.07	7.021 (0.008)*	74.522 (<0.001)*	0.499 (0.480)
TMTb-a time (s)	116.51 ± 60.34	100.33 ± 43.67	133.38 ± 65.05	142.81 ± 70.27	4.781 (0.029)*	59.246 (<0.001)*	0.469 (0.494)

Values are mean ± standard deviation. The comparisons of gender and *APOE* ε4 status were performed using Wald chi-square test. Multivariable analysis of covariance was used to determine the interactive effect of rs541458 × age and the main effects of rs541458 and age on the neuropsychological tests (gender, years of education, and *APOE* ε4 status as covariates). *P* < 0.05 was considered significant

*PICALM* phosphatidylinositol-binding clathrin assembly protein, *APOE* apolipoprotein E, *MMSE* Mini-Mental State Examination, *AVLT* Auditory Verbal Learning Test, *ROCF* Rey-Osterrieth Complex Figure, *CDT* Clock-Drawing Test, *CVFT* Category Verbal Fluency Test, *BNT* Boston Naming Test, *SDMT* Symbol Digit Modalities Test, *TMT* Trail Making Test, *SCWT* Stroop Color and Word Test

*P < 0.05

### Neuropsychological Testing

All the participants were subjected to a battery of neuropsychological tests that assessed several cognitive domains. As mentioned previously, the comprehensive neuropsychological battery comprised the following six cognition domains (the tests used to assess each domain are in parentheses): 1, general mental status (the Mini-Mental Status Examination—Chinese version (MMSE) [41]); 2, memory function (the Auditory Verbal Learning Test (AVLT) [42], the Rey-Osterrieth Complex Figure test (ROCF) (recall) [43], and the Digit Span test, which was a sub-test of the Wechsler Adult Intelligence Scale—Chinese revision); 3, spatial processing (ROCF-copy [43] and the Clock-Drawing Test (CDT) [44]); 4, language (the Category Verbal Fluency Test (CVFT) and the Boston Naming Test (BNT) [45]); 5, processing speed (the Trail Making Test (TMT) A [46] and the Symbol Digit Modalities Test (SDMT) [47]); and 6, executive function (the TMT-B [46] and the Stroop Color and Word Test C (SCWT) [48]).

### Analysis of Genotyping

*PICALM* rs541458 was genotyped using TaqMan allele-specific assays on the 7900HT Fast Real-Time PCR System (Applied Biosystems, Foster City, CA, USA). Another two SNPs, rs429358 and rs7412, which collectively form the *APOE* ε2 (with the haplotype of rs429358-rs7412: T/T), *APOE* ε3 (G/T), and *APOE* ε4 alleles (G/G), were also genotyped. The sample success rate for all three SNPs were 100% (i.e., no failures across the participants to “call” the polymorphisms), and the reproducibility of all of the genotyping was 100% according to a duplicate analysis of 10% of the genotypes. Given the risk of harboring the T allele, we combined the CT and TT genotypes into T allele carriers. Thus, according to the rs541458 genotyping, all participants were divided into two groups: 151 CC and 487 T allele carriers.

### MRI Data Acquisition

To investigate the interactive effects between age and *PICALM* genotypes on brain structure and function, we also acquired MRI data from a sub-cohort (*n* = 78) of the study participants using a SIEMENS TRIO 3T scanner in the Imaging Center for Brain Research, Beijing Normal University. The participants were placed in a supine position, with their head snugly fixed by straps and foam pads to minimize head movement. Resting state data were collected using a gradient echo EPI sequence [TE = 30 ms, TR = 2000 ms, flip angle = 90°, 33 slices, slice thickness = 4 mm, in-plane matrix = 64 × 64, field of view = 256 × 256 mm^2^]. During the single-run resting acquisition, the subjects were instructed to remain awake, relaxed, and with their eyes closed and to remain as motionless as possible. The resting acquisition lasted for 8 min, and 240 image volumes were obtained. T1-weighted structural images were acquired using 3D magnetization prepared rapid gradient echo (MP-RAGE) sequences [176 sagittal slices, TE = 3.44 ms, TR = 1900 ms, flip angle = 9°, slice thickness = 1 mm, acquisition matrix = 256 × 256, field of view = 256 × 256 mm^2^]. Table 2 provides further details of the imaging sub-sample.

**Table 2:** Demographics and neuropsychological test results of imaging sub-sample

	Age <65 (*N* = 41)		Age ≥65 (*N* = 37)		*F*/*χ* ^2^_age × *PICALM*_	*F*/*χ* ^2^_age_	*F*/*χ* ^2^_*PICALM*_
	*PICALM* CC (*N* = 18)	*PICALM* T carriers (*N* = 23)	*PICALM* CC (*N* = 15)	*PICALM* T carriers (*N* = 22)	(*P*)	(*P*)	(*P*)
Sex (male/female)	7/11	11/12	8/7	9/13	0.869 (0.351)	0.890 (0.346)	0.726 (0.394)
Education	8.83 ± 2.36	9.48 ± 2.15	11.93 ± 3.15	10.36 ± 3.87	2.643 (0.108)	8.560 (0.005)*	0.461 (0.499)
*APOE* ε4 or not	3/15	4/19	3/12	8/14	0.457 (0.499)	0.081 (0.776)	0.153 (0.696)
General mental status							
MMSE	27.11 ± 1.81	27.35 ± 2.04	27.80 ± 1.61	25.50 ± 2.20	6.498 (0.013)*	1.111 (0.295)	4.363 (0.040)*
Memory function							
AVLT-delay	4.22 ± 2.96	4.22 ± 2.61	3.67 ± 2.32	3.14 ± 2.75	0.018 (0.893)	2.098 (0.152)	0.055 (0.815)
AVLT-total	24.00 ± 9.89	25.43 ± 8.46	24.20 ± 8.42	22.00 ± 9.25	0.402 (0.528)	0.633 (0.429)	0.002 (0.964)
ROCF-delay	13.72 ± 5.95	12.17 ± 7.08	12.47 ± 6.90	7.73 ± 5.76	0.635 (0.428)	4.457 (0.038)*	3.869 (0.053)
Forward digit span	7.17 ± 1.15	7.00 ± 1.38	6.60 ± 1.18	7.32 ± 1.25	2.347 (0.130)	0.046 (0.831)	0.996 (0.322)
Backward digit span	4.22 ± 1.00	4.52 ± 1.50	4.27 ± 1.44	4.14 ± 1.21	0.304 (0.583)	0.588 (0.446)	0.056 (0.814)
Spatial processing							
ROCF-copy	33.11 ± 2.03	33.04 ± 3.24	31.60 ± 8.98	32.09 ± 3.61	0.923 (0.340)	3.705 (0.058)	0.208 (0.650)
CDT	24.61 ± 3.79	24.70 ± 3.51	25.80 ± 2.04	23.36 ± 4.16	0.897 (0.347)	1.144 (0.288)	1.551 (0.217)
Language							
CVFT	38.11 ± 7.24	44.13 ± 8.46	43.87 ± 8.97	41.27 ± 11.98	3.158 (0.080)	0.091 (0.763)	0.673 (0.415)
BNT	22.83 ± 4.41	23.83 ± 3.42	23.67 ± 2.87	21.45 ± 3.81	0.889 (0.349)	3.161 (0.080)	0.085 (0.772)
Processing speed							
SDMT	29.22 ± 8.63	34.87 ± 9.30	33.13 ± 11.29	25.64 ± 7.49	6.769 (0.011)*	3.670 (0.059)	0.047 (0.829)
TMTa time (s)	70.72 ± 33.09	57.52 ± 27.94	65.60 ± 26.01	79.64 ± 36.64	3.473 (0.067)	0.591 (0.445)	0.001 (0.970)
Executive function							
SCWT C-B time (s)	44.78 ± 18.62	35.96 ± 14.79	41.00 ± 21.10	48.18 ± 24.65	4.700 (0.034)*	0.372 (0.544)	0.016 (0.901)
SCWT-C time (s)	86.94 ± 22.39	77.35 ± 21.38	81.47 ± 24.86	93.36 ± 25.68	5.354 (0.024)*	0.680 (0.412)	0.200 (0.656)
TMTb time (s)	199.33 ± 78.13	148.35 ± 43.08	187.93 ± 76.69	246.73 ± 67.88	9.211 (0.003)*	10.452 (0.002)*	0.003 (0.954)
TMTb-a time (s)	128.61 ± 55.24	90.83 ± 34.18	122.33 ± 67.30	167.09 ± 64.07	6.770 (0.011)*	12.224 (0.001)*	0.002 (0.961)

Values are mean ± standard deviation. The comparisons of gender and *APOE* ε4 status were performed using Wald chi-square test. Multivariable analysis of covariance was used to determine the interactive effect of rs541458 × age and the main effects of rs541458 and age on the neuropsychological tests (gender, years of education, and *APOE* ε4 status as covariates). *P* < 0.05 was considered significant

*PICALM* phosphatidylinositol-binding clathrin assembly protein, *APOE* apolipoprotein E, *MMSE* Mini-Mental State Examination, *AVLT* Auditory Verbal Learning Test, *ROCF* Rey-Osterrieth Complex Figure, *CDT* Clock-Drawing Test, *CVFT* Category Verbal Fluency Test, *BNT* Boston Naming Test, *SDMT* Symbol Digit Modalities Test, *TMT* Trail Making Test, *SCWT* Stroop Color and Word Test

*P < 0.05

### Data Processing and Analysis

#### Resting state image preprocessing and analysis

For each participant, the first 10 volumes were discarded to allow the participants to adapt to the magnetic field. Functional data were preprocessed using SPM and DPARSF (http://rfmri.org/DPARSF), and the processing included slice timing, within-subject inter-scan realignment to correct for possible movement, spatial normalization to a standard brain template in the Montreal Neurological Institute (MNI) coordinate space, resampling to 3 × 3 × 3 mm^3^, and smoothing with an 8 mm full-width half-maximum Gaussian kernel. Three subjects were excluded because of unacceptable head movement (translation >3 mm or rotation >3°) during the fMRI scanning.

We then performed the independent component analysis (ICA) using the group ICA toolbox (GIFT version 2.0e; http://mialab.mrn.org/software/gift/). Twenty-five components were estimated for each subject. Three main stages were used when applying the ICA to all participants: (i) principal component analysis was performed for each subject for data reduction, (ii) application of the ICA algorithm, and (iii) back-reconstruction for each individual subject. After back-reconstruction, the mean spatial maps of each group at every time point were converted to *z*-scores for display. We focused on the DMN, left frontal-parietal network (FPN), and right FPN in the current study. The best-fit components for the three resting state networks were identified by visual inspection. For each network, a full factorial analysis of covariance (ANCOVA) (2 × 2) was conducted with *PICALM* rs541458 (T carriers versus CC genotype) and age (<65 years old versus ≥65 years old) as independent factors in SPM 8 (Statistical Parametric Mapping, www.fil.ion.ucl.ac.uk/spm) with gender, years of education, and *APOE* ε4 status included as covariates (*p* < 0.05, AlphaSim-corrected). Additionally, further analysis of the correlation of imaging measures with neuropsychological tests was performed on any significant clusters resulting from the voxel-wise comparisons. For each significant cluster, the connectivity values were extracted by averaging the intensities over all voxels within the cluster from every participant’s component map.

#### Structural Image Analysis

We used a voxel-based morphometry (VBM) analysis to compare the whole-brain gray matter volume of all subjects using the VBM8 software package (http://dbm.neuro.uni-jena.de/vbm/). The preprocessing of structural images used default parameters, except for the estimation using “ICBM space template—East Asian brains” and extended options using “thorough cleanup.” The images were bias-corrected, tissue classified, and normalized to Montreal Neurological Institute space using affine and non-linear transformations to compare the absolute amounts of tissue [49]. One subject with excessive head motion was identified and excluded. The images of the remaining 77 subjects were free of such problems. The modulated gray matter images were smoothed with a Gaussian kernel of 8 mm full width at half maximum (FWHM). We utilized the mean gray matter map (threshold = 0.3) of all subjects to obtain a group-based brain mask and used it for subsequent analysis. A full factorial ANCOVA (2 × 2) was calculated using SPM8 with *PICALM* rs541458 (T carriers versus CC genotype) and age (<65 years old versus ≥65 years old) as independent factors with gender, years of education, *APOE* ε4 status, and total intracranial volume included as covariates (*p* < 0.001, AlphaSim-corrected). The significant areas for the interaction of rs541458 × age in the VBM analysis were extracted as regions of interest (ROI) using REST v1.8 (http://www.restfmri.net).

### Statistical Analysis

The Hardy-Weinberg test was completed using the PLINK software [50]. For the demographic factors of gender and years of education and the *APOE* ε4 status, the multivariable analysis of variance or Wald chi-square test was used to assess the *PICALM* rs541458 polymorphism and age effects. For the neuropsychological assessments, a multivariable analysis of covariance was conducted, with gender, years of education, and *APOE* ε4 status as covariates. In addition, for the functional connectivity, gray matter volume, and cognitive performance showing the significant interactive effects of rs541458 × age, we further calculated the correlation between them using Pearson partial correlation analyses, after controlling for the influences of gender, years of education, and *APOE* ε4 status in the four genotype and age groups, separately.

## Results

### Demographic and Neuropsychological Results

The rs541458 SNP did not show any deviations from Hardy-Weinberg equilibrium in all participants (*P* > 0.05). For all participants, the differences between the rs541458 groups and the rs541458 by age interactions were not significant for demographic factors or *APOE* ε4 status (Table 1). These results were the same in the imaging sub-sample (Table 2). All subsequent analyses were adjusted for gender, years of education, and *APOE* ε4 status.

For the neuropsychological tests, the interactions of rs541458 × age were significant for executive function (SCWT-C, *P* = 0.008, TMTb, *P* = 0.008, and TMTb-a, *P* = 0.029) and processing speed (TMTa, *P* = 0.028) in all participants. The effects of age, but not rs541458, were significant for all neuropsychological tests in all participants (Table 1). We found similar results in the imaging sub-sample, as the interactions of rs541458 × age were significant for executive function (SCWT C-B, *P* = 0.034, SCWT-C, *P* = 0.024, TMTb, *P* = 0.003, and TMTb-a, *P* = 0.011), processing speed (SDMT, *P* = 0.011), and general mental status (MMSE, *P* = 0.013). The age effects were significant for executive function (TMTb, *P* = 0.002, and TMTb-a, *P* = 0.001) and memory function (ROCF-delay, *P* = 0.038). The rs541458 effects were significant for general mental status (MMSE, *P* = 0.040) (Table 2, Fig. S1).

### Interactive Effect of rs541458 × age on Resting State Networks

**Fig. 1:**
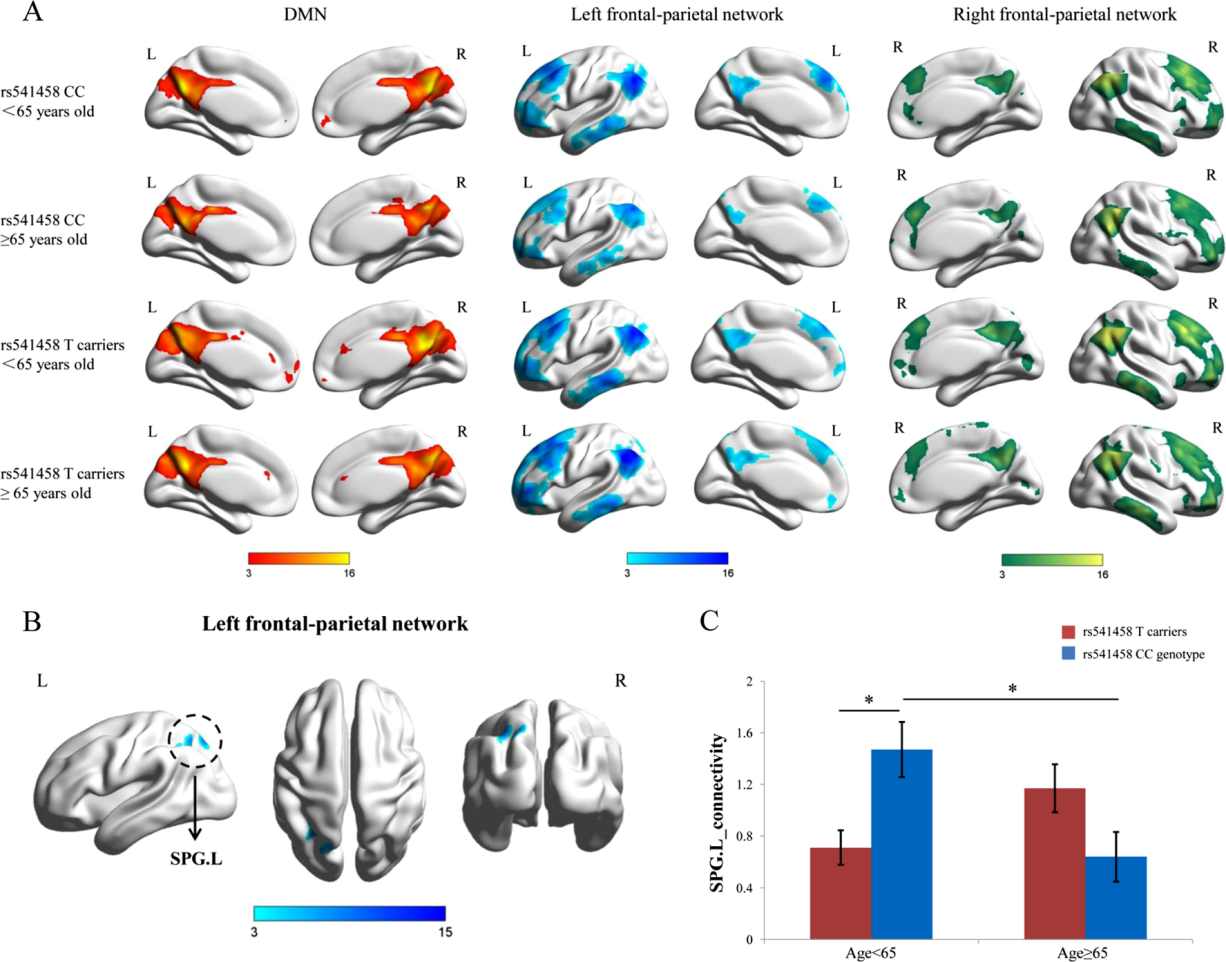
**a** The spatial maps show the DMN, left FPN, and right FPN of the four genotype and age groups, separately. **b** The brain map represents voxel-wise interactive effect of rs541458 × age on the left FPN (*SPG.L x* = −18 mm, *y* = −75 mm, *z* = 54 mm; voxel size = 73, *P* < 0.05, AlphaSim-corrected). The *x*, *y*, *z* coordinates of the primary peak in MNI space. **c** The bar graph shows the ROI analysis on the significant regions from voxel-wise comparisons. *Error bars* denote the standard error of the mean. *Significant at *P* < 0.05. *DMN* default mode network, *FPN* frontal-parietal network, *SPG.L* left superior parietal gyrus, *ROI* region of interest

For the resting state networks, the DMN, left FPN, and right FPN were identified from the results of the group ICA (Fig. 1a). The full factorial analysis of covariance (ANCOVA) (2 × 2) revealed a significant interaction effect of rs541458 × age on the brain region connectivity of the left superior parietal gyrus (SPG.L) (*x* = −18 mm, *y* = −75 mm, *z* = 54 mm; voxel size = 73, *P* < 0.05, AlphaSim-corrected, Fig. 1b) within the left FPN. The simple effects of rs541458 and age were further analyzed (Fig. 1c). The functional connectivity of the SPG.L in the T allele carriers <65 years old group was significantly lower than that in the CC genotype <65 years old group (*F* = 8.56, *P* = 0.005). Further, the functional connectivity of the SPG.L in the CC genotype ≥65 years old group was significantly lower than that in the CC genotype <65 years old group (*F* = 9.35, *P* = 0.003).

### Interactive Effect of rs541458 × age on Gray Matter Volumes

**Fig. 2:**
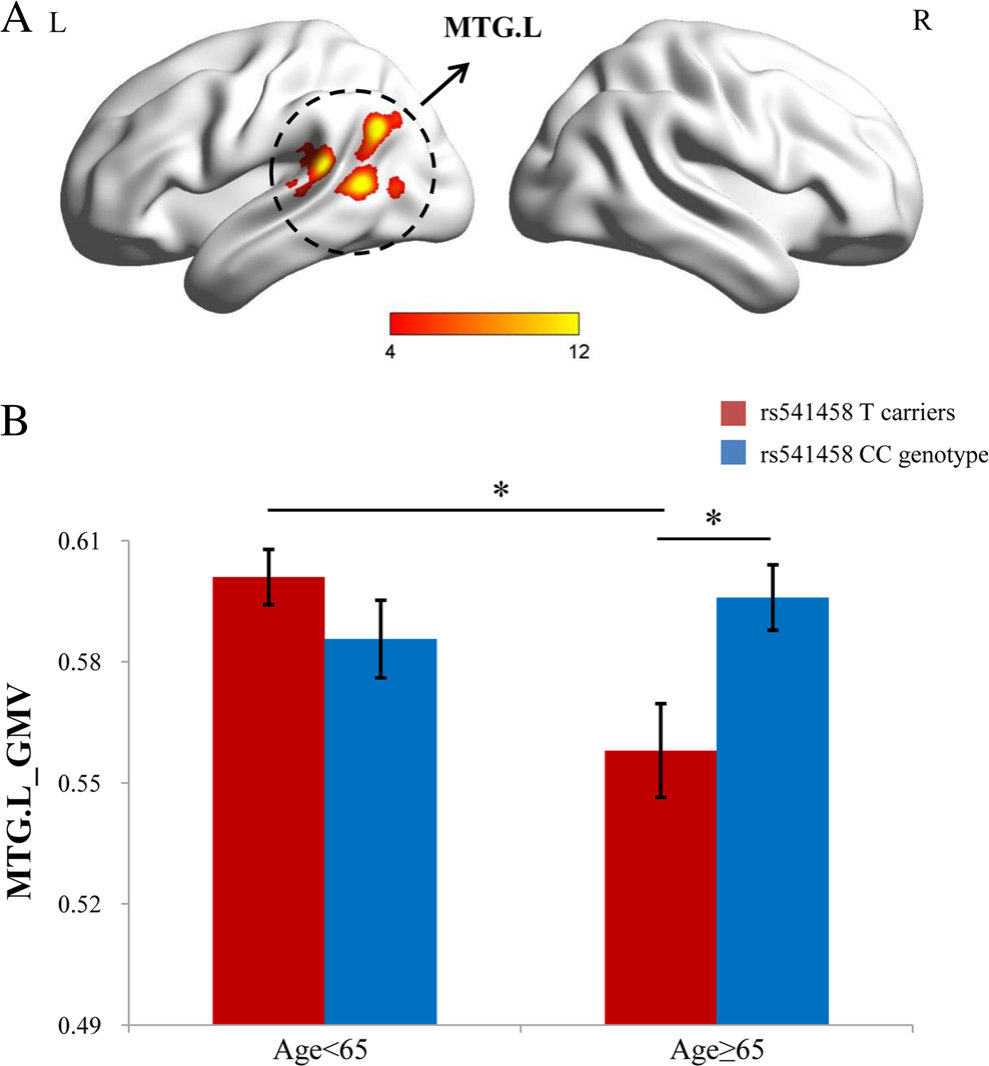
**a** The brain map represents voxel-wise interactive effect of rs541458 × age on the gray matter volumes (*MTG.L x* = −50 mm, *y* = −51 mm, *z* = 4 mm; voxel size = 3032, *P* < 0.001, AlphaSim-corrected). The *x*, *y*, *z* coordinates of the primary peak in MNI space. **b** The bar graph shows the ROI analysis on the significant regions from voxel-wise comparisons. *Error bars* denote the standard error of the mean. *Significant at *P* < 0.05. *MTG.L* left middle temporal gyrus, *ROI* region of interest

We found a significant interaction effect of rs541458 × age on the gray matter volume of the left middle temporal gyrus (MTG.L) (*x* = −50 mm, *y* = −51 mm, *z* = 4 mm; voxel size = 3032, *P* < 0.001, AlphaSim-corrected, Fig. 2a). The simple effects of rs541458 and age were further analyzed (Fig. 2b). The gray matter volume of the MTG.L in the T allele carriers <65 years old group was significantly higher than that in the T allele carriers ≥65 years old group (*F* = 11.70, *P* = 0.001). And the gray matter volume of the MTG.L in the CC genotype ≥65 years old group was significantly higher than that in the T allele carriers ≥65 years old group (*F* = 8.69, *P* = 0.004).

### Correlations Among Functional Connectivity, Gray Matter Volume, and Cognitive Performance

**Fig. 3:**
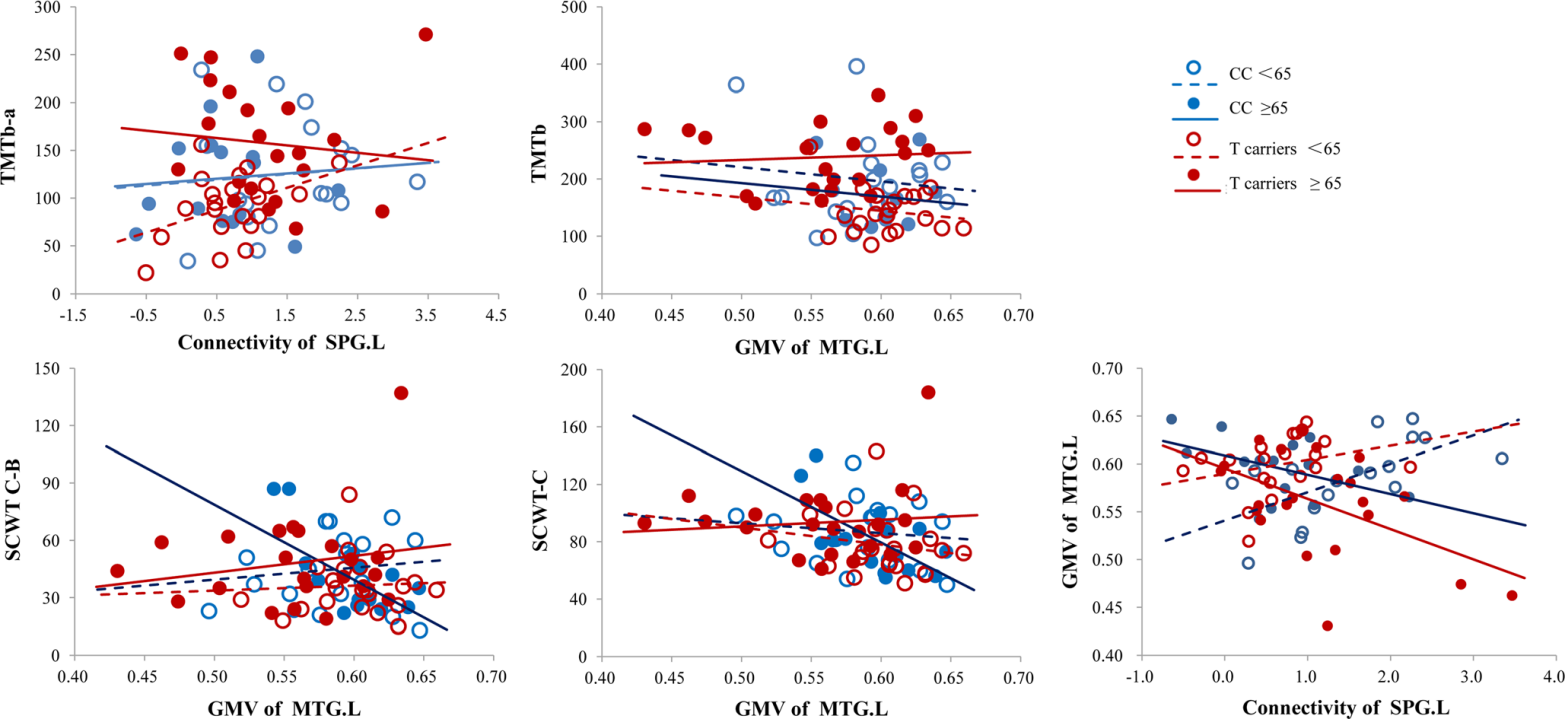
Correlations between the functional connectivity of SPG.L, the gray matter volume of MTG.L, and the neuropsychological tests in the four genotype and age groups, separately. The significant correlation showed between the connectivity of SPG.L and the TMTb-a time in T allele carriers <65 years old group, between the volume of MTG.L and the TMTb time in CC genotype <65 years old group, between the volume of MTG.L and the SCWT C-B and SCWT-C time in the CC genotype ≥65 years old group, and between the connectivity of SPG.L and the volume of MTG.L in the CC genotype ≥65 years old group. *SPG.L* left superior parietal gyrus, *MTG.L* left middle temporal gyrus, *TMT* Trail Making Test, *SCWT* Stroop Color and Word Test

To assess whether the functional network and gray matter volume differences could explain the cognitive performance differences, we correlated the connectivity of the SPG.L within the left FPN and the gray matter volume of the MTG.L each with the cognitive test score, which also showed significant rs541458 × age interactive effects. For the functional network, the T allele carriers <65 years old group showed a significant correlation between TMTb-a and SPG.L connectivity (*r* = 0.460, *P* = 0.047) (Fig. 3). For the gray matter volume, the TMTb was negatively associated with MTG.L volume in the CC genotype <65 years old group (*r* = −0.588, *P* = 0.017), and SCWT was negatively associated with MTG.L volume in the CC genotype ≥65 years old group (SCWT C-B, *r* = −0.607, *P* = 0.028, SCWT-C, *r* = −0.635, *P* = 0.020) (Fig. 3). No significant correlations were found in the other groups or for the other cognitive performance measures. Furthermore, the CC genotype ≥65 years old group showed a negative correlation between SPG.L connectivity and MTG.L volume (*r* = −0.652, *P* = 0.022) (Fig. 3). Notably, there were no correlation results that survived (*P* < 0.05) after FDR correction for multiple comparisons.

## Discussion

The findings from the present study showed that age modulated the effects of *PICALM* on cognitive performance, which were mainly manifested in the executive function and processing speed, that is, the risk allele T was associated with better cognitive performance in the <65 years old group, but the opposite was found in the ≥65 years old group. The effects of *PICALM* on resting state functional networks were also modulated by age. We found that the risk allele T was associated with decreased functional connectivity of the SPG.L within the left FPN in the <65 years old group, whereas the T allele was associated with increased connectivity in the ≥65 years old group. Age also showed a modulatory effect on the association between *PICALM* and the gray matter volume of the MTG.L. In further correlation analyses, the functional connectivity of the SPG.L was positively correlated with TMTb-a time in the T allele carriers <65 years old group, which indicated that lower functional connectivity was linked to better executive function. Both CC genotype groups showed that an increased volume of the MTG.L was significantly related to better executive function.

First, we found that the age difference could affect the relations between the rs541458 T allele and both executive function and processing speed. With the exception observed in the study by Chen et al. [21] for Asian ethnic groups, several previous studies reported the T allele of rs541458 as a risk factor for LOAD in westerners [18–20]. However, few studies have directly investigated the association between rs541458 and cognitive functions, and none have been conducted on the rs541458 and age interaction. Indirectly, limited evidence has demonstrated that the rs541458 T allele would accelerate the decreases of general cognitive functions, thus suggesting the influence of age on the association between rs541458 and cognitive functions [26]. Implicitly, this was similar to our results, which indicated that age would modulate the effects of rs541458 on cognition. Interestingly, the risk G allele of another *PICALM* polymorphism, rs3851179, was associated with general cognition impairments in >70-year-old Parkinson’s disease patients but not in ≤70-year-old patients [51]. Further, the protective A allele of rs3851179 was associated with better cognitive composite scores only in men of the oldest elderly group [52]. These results together suggested that when the *PICALM* polymorphism is acting on cognition, other factors such as age would play regulatory effects. To further investigate the *PICALM* by age interaction, we further evaluated both the brain functional networks and its structure.

Ample data suggest that the FPN is sensitive to damage in mild cognitive impairment (MCI), which is a precursor phase of AD [53, 54]. A previous study of the *APOE* genotype found an intrinsic effect of *APOE* on the functional architecture of FPN [36]. In our study, the association between rs541458 and the functional connectivity of the left FPN was affected by age, and the pattern was contrary to that found for cognitive functions. One possible explanation for this difference is a compensation mechanism of functional networks. It is noteworthy that the FPN is essential for higher cognitive behaviors and that the decreases of effective connectivity in frontal-parietal circuits made for impairments in top-down attention control [55]. Recent studies have revealed the *PICALM* rs3851179 and clusterin/apolipoprotein J protein gene (*CLU*) interactions on functional connectivity, verified both in Caucasian and Chinese subjects [37, 56]. Nevertheless, the effects of rs541458 on functional networks and the role of age are still poorly understood. Pooling those previous studies and our current results would suggest, to some extent, that the effects of *PICALM* on brain functions are influenced not only by other genetic factors but also by age. Further, Wang et al. reported a decreased functional connectivity between the superior parietal cortex and thalamus in MCI patients [57], which may suggest the sensitivity of connectivity in the SPG for cognitive impairments.

Consistent with our brain connection findings, we also found that the effects of rs541458 on gray matter volume of the MTG.L were modulated by age. The MTG was among the first regions to show structural changes in amnestic MCI [58] and AD patients [59]. Compared with non-converters, MCI converters showed greater gray matter losses in the MTG area [60]. This may result from elevated tau pathology and neuronal loss [61]. In an elderly European group, the risk G allele of rs3851179 was related to a smaller gray matter volume of the inferior frontal gyrus compared to the protective A allele in *APOE* ε4 allele carriers, but this was not observed in non-carriers [39]. Similarly, *CLU* could affect the association between rs3851179 and hippocampal volume; that is, reversed patterns were shown in the *CLU* risk and protective groups [56]. This was consistent with our results, which showed that the risk allele T was associated with a higher gray matter volume of the MTG.L in the <65 years old group and with a lower volume in the ≥65 years old group.

Interestingly, the interactive effects of rs541458 × age on resting state functional networks and gray matter volume were both located on the left hemisphere, probably suggesting that lateralization were associated with aging and dementia. A recent research showed that age is strongly related to lateralization in multiple regions within the frontal network, attentional network, sensorimotor network, and visual network [62]. Early research has suggested that some neurodegenerative disease like dementia exhibited injury primarily in the left rather than the right hemisphere, and hypometabolism is more susceptible to neurodegeneration in the left hemisphere [63]. Some studies also demonstrated that brain asymmetry can be observed at biochemical level like hippocampal nitric oxide mediator system [64].

A growing body of research has demonstrated that particular cognitive impairments are closely related to the disconnection of brain networks. This has been verified in several neurodegenerative diseases, including AD [65] and frontotemporal dementia [66]. TMT is a frequently used neuropsychological test for executive function [46]. The executive function depended on disrupted regions to collaborate, mainly including the left SPG and lateral prefrontal cortex [67]. In MCI patients, the white matter hyperintensities in the FPN were associated with decreased executive function [68]. Our study found that the functional connectivity of the SPG.L was positively related with TMTb-a time scores in T allele carriers <65 years old, which probably suggests that better executive function performance did not need higher connectivity for support. Similarly, recent results have shown that increased connectivity between the left parietal and middle temporal cortices was associated with decreased global cognitive status in cognitively normal elderly [69]. The connectivity between the left inferior parietal and medial prefrontal cortex was related to episodic memory performance, which has also been found in cognitively normal older adults [70]. Furthermore, we found that the increased volume of the MTG.L was related to better executive function in both CC genotype groups. In the *APOE* ε4 carriers, the apparent diffusion coefficient of the temporal lobe could predict executive function [71]. In probable AD patients, decreased metabolism in the temporal cortex was correlated with poor performance on executive functions [72]. Additionally, a previous study indicated strong correlations between medial temporal lobe atrophy and executive functions in non-demented elderly [73]. As corollaries, some brain function- and structure-related measures, such as the functional connectivity of resting state networks and gray matter volumes, could be biomarkers for predicting variants in cognitive functions.

As mentioned above, abnormal PICALM expression would disturb APP metabolism [74] due to the crucial role of PICALM in clathrin-mediated endocytosis [12]. PICALM would promote the transportation of APP-cleaved C-terminal fragment (APP-CTF) from the plasma membrane intracellularly to allow fusion with autophagosomes and endosomes, thus increasing the degradation of APP-CTF and decreasing the production of amyloid-β (Aβ) [75]. Meanwhile, the *PICALM* rs541458 T allele is associated with decreased cerebrospinal fluid Aβ_42_ [76]. Considering its high expression in endothelial cells [77], this finding may imply that PICALM plays a major role in removing Aβ from the brain through the blood-brain barrier, supported by a close relationship between high levels of PICALM and increased Aβ clearance [78]. Moreover, the expression of PICALM was reduced and co-localized with hyperphosphorylated tau in AD patients [79], possibly indicating a role of PICALM in tauopathy [80]. Thus, one might speculate that an abnormal level of PICALM would contribute to the dysfunction of endocytosis and a series of relevant pathological changes related to AD. However, the exact mechanisms by which rs541458 contributes to AD etiology are yet to be confirmed in animal and human studies.

The present study had some limitations. First, the effects of different social class or level of educational achievement should be noted that they may have different effects on cognitive function and brain. Second, it is very important to validate the present results by other Chinese cohort and some longitudinal studies. Third, the significant correlations between neuroimaging measurements and cognitive performances reported in the present study should be regarded as exploratory in nature due to no correlation survived (*P* < 0.05) after FDR correction for multiple comparisons. Thus, they need to be confirmed in future additional studies. Overall, the findings of this study should be interpreted with these limitations in mind.

In summary, the present study suggested the modulation of age on the association of the *PICALM* rs541458 polymorphism with executive function, on its association with the left FPN and the MTG.L volume, and on the associations between neuropsychological performance and brain connection/structure in non-demented Chinese elderly. This finding highlights the importance of combining age and genetic polymorphisms when examining candidate genes that affect cognitive function. Further studies with a larger sample size and longitudinal design are needed to confirm our results.

## Electronic supplementary material

Fig. S1 Bar graphs show significant interactions of rs541458×age in the neuropsychological assessments in the imaging sub-sample. Error bars denote the standard error of the mean. *Significant at *P*<0.05. MMSE, Mini-Mental State Examination; SDMT, Symbol Digit Modalities Test; TMT, Trail Making Test; SCWT, Stroop Color and Word Test. (GIF 77 kb)

High Resolution Image (TIFF 15190 kb)
