# Supplementary figures and images for: The Interactive Effects of Age and PICALM rs541458 Polymorphism on Cognitive Performance, Brain Structure, and Function in Non-demented Elderly
Source: Mol Neurobiol. 2017 Jan 23;55(2):1271–83. doi: 10.1007/s12035-016-0358-5 (PMC5820373; doi:10.1007/s12035-016-0358-5)

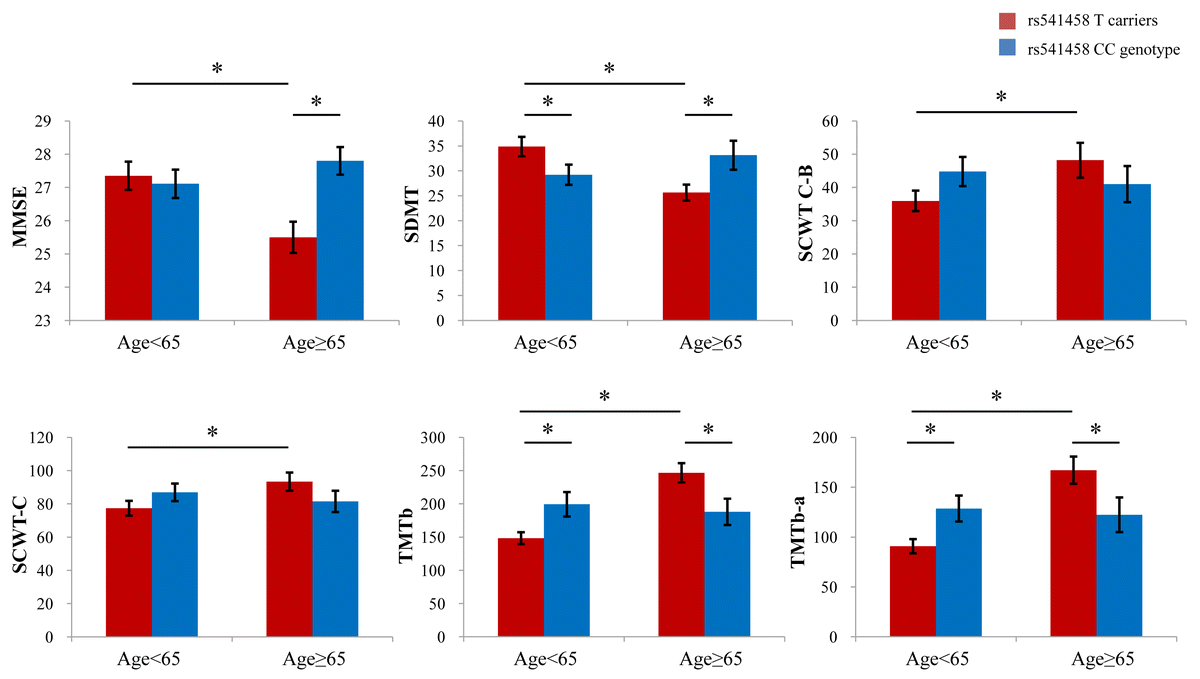

Supplement: Supplementary file 1 — Bar graphs show significant interactions of rs541458×age in the neuropsychological assessments in the imaging sub-sample. Error bars denote the standard error of the mean. *Significant at P<0.05. MMSE, Mini-Mental State Examination; SDMT, Symbol Digit Modalities Test; TMT, Trail Making Test; SCWT, Stroop Color and Word Test. (GIF 77 kb) [file 12035_2016_358_Fig4_ESM.gif]

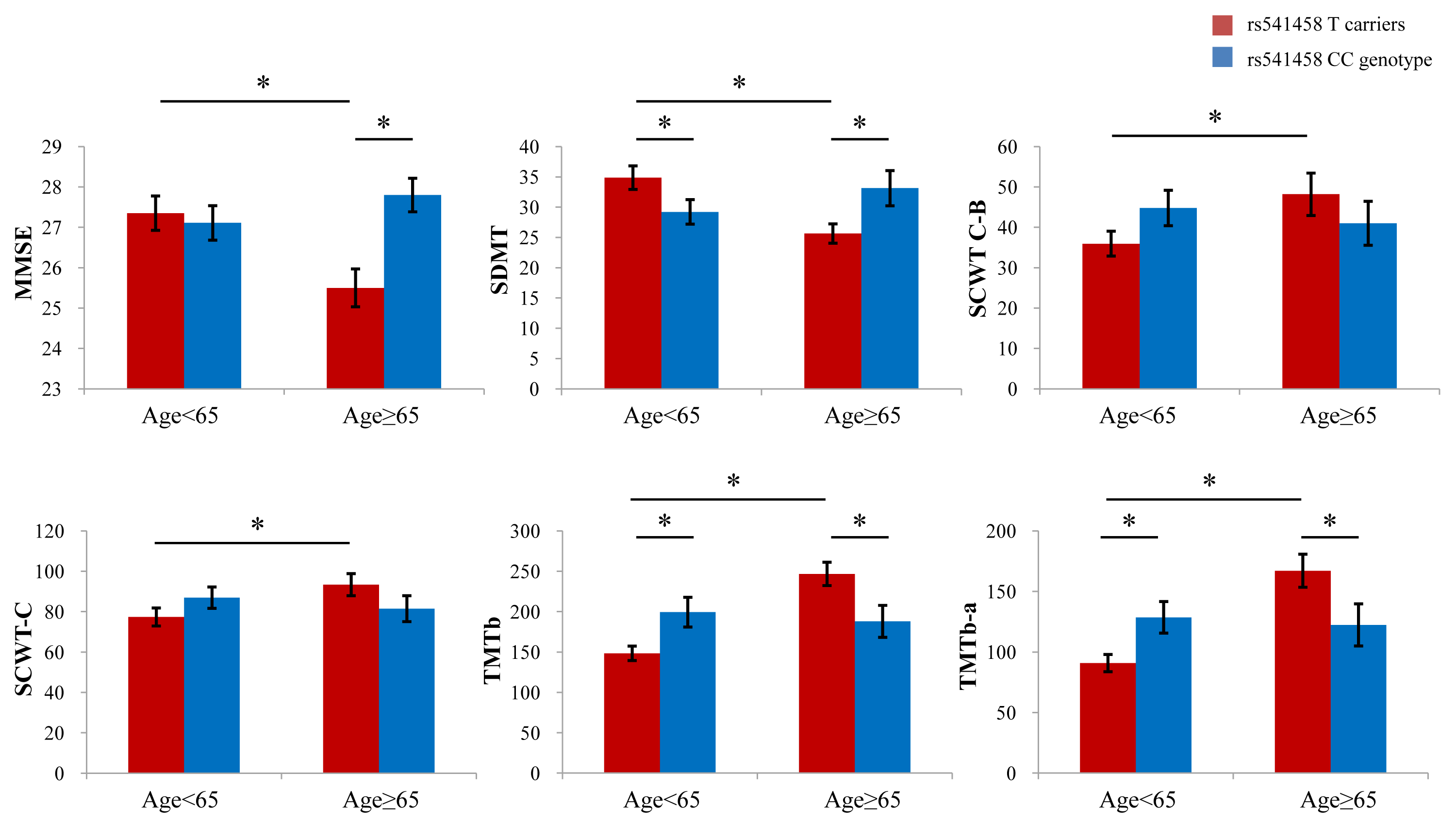

Supplement: Supplementary file 2 — (TIFF 15190 kb) [file 12035_2016_358_MOESM1_ESM.tif]
